# Charge Redistribution of Co_9_S_8_/MoS_2_ Heterojunction Microsphere Enhances Electrocatalytic Hydrogen Evolution

**DOI:** 10.3390/biomimetics8010104

**Published:** 2023-03-05

**Authors:** Lili Zhang, Jitang Zhang, Aijiao Xu, Zhiping Lin, Zongpeng Wang, Wenwu Zhong, Shijie Shen, Guangfeng Wu

**Affiliations:** 1 College of Material Science and Engineering, Changchun University of Technology, Changchun 130051, China; 2 Zhejiang Provincial Key Laboratory for Cutting Tools, Taizhou University, Jiaojiang 318000, China

**Keywords:** heterojunction, thin nanosheets, Co_9_S_8_, MoS_2_, electrocatalyst, hydrogen evolution reaction

## Abstract

The electrocatalytic hydrogen evolution activity of transition metal sulfide heterojunctions are significantly increased when compared with that of a single component, but the mechanism behind the performance enhancement and the preparation of catalysts with specific morphologies still need to be explored. Here, we prepared a Co_9_S_8_/MoS_2_ heterojunction with microsphere morphology consisting of thin nanosheets using a facile two-step method. There is electron transfer between the Co_9_S_8_ and MoS_2_ of the heterojunction, thus realizing the redistribution of charge. After the formation of the heterojunction, the density of states near the Fermi surface increases, the *d*-band center of the transition metal moves downward, and the adsorption of both water molecules and hydrogen by the catalyst are optimized. As a result, the overpotential of Co_9_S_8_/MoS_2_ is superior to that of most relevant electrocatalysts reported in the literature. This work provides insight into the synergistic mechanisms of heterojunctions and their morphological regulation.

## 1. Introduction

With the increase in global population and rapid economic development, environmental degradation and the energy issue are starting to gain attention, and this created a demand for new clean and efficient energy sources. Considering its renewable attributes and high energy density, hydrogen is one of the most promising green energy sources [1,2,3]. There are four main ways to produce hydrogen: from fossil fuels, from industrial by-products, from biomass, and from the electrolysis of water [4]. The first three of these require unsustainable sources of raw materials. Hydrogen production from electrolytic water splitting has gained worldwide attention because of its environmental friendliness, production flexibility, and product purity [5,6,7]. Efficient electrolytic hydrogen production from water requires effective electrocatalysts to reduce the energy consumption of the hydrogen evolution reaction (HER). Pt-based materials are benchmark catalysts for the HER, but their high cost and scarcity hinder large-scale commercial applications [8,9,10]. Therefore, it is crucial to design electrocatalysts that are both affordable and high-performing in order to enable the development of hydrogen energy.

In recent years, materials based on transition metal sulfides have been considered as potential alternatives to noble metal catalysts due to their low cost, high activity, and stability [11,12,13,14]. Density functional theory calculations indicate that MoS_2_ has a ΔG_H*_ value close to that of Pt, demonstrating that it may be a promising HER electrocatalyst [15]. Subsequent electrochemical characterization has confirmed that MoS_2_ does indeed have excellent HER activity [16]. In fact, MoS_2_ has many different phases [17]. Among them, the H and T phases, in which Mo atoms are located at the centers of triangular prisms and octahedra, respectively, are common. Because 2H-MoS_2_ is semiconducting and 1T-MoS_2_ is metallic, 1T-MoS_2_ is usually considered suitable as an electrocatalyst. Co_9_S_8_ was also explored as a HER electrocatalyst many years ago because of its excellent metal conductivity [18]. By controlling the morphology and compounding with a conductive substrate, the HER properties of Co_9_S_8_-based materials have been greatly improved and have attracted a lot of attention [19,20,21]. Although much progress has been made, the low intrinsic activity of a single sulfide is still a factor limiting its application. The construction of a heterojunction of two transition metal sulfides can combine their respective characteristics and realize charge redistribution, which is conducive to improving overall electrocatalytic performance [22]. In addition, by controlling the morphology of the catalyst, more active sites can be exposed as much as possible and participate in the electrocatalytic reaction [17]. Up to now, a large number of Co_9_S_8_/MoS_2_-related heterojunctions have been explored as HER electrocatalysts [23,24,25]. Although the HER performance has been greatly improved in each of these heterojunctions compared with that of the individual components, the mechanism behind the enhanced performance and the preparation of catalysts with specific morphologies remains to be explored.

Herein, we prepared a Co_9_S_8_/MoS_2_ heterojunction with microsphere morphology consisting of thin nanosheets using a facile two-step reaction. There are many pores between the nanosheets which help to increase the surface area and promote the migration of electrolytes and bubbles. There is obvious electron transfer between the Co_9_S_8_ and MoS_2_ of the heterojunction, thus realizing the redistribution of charge. The formation of heterojunctions shifts the *d*-band centers of both the Co and Mo downward, thus weakening the adsorption of hydrogen by the catalyst and achieving closer to zero ΔG_H*_. In addition, the increase in the density of states near the Fermi surface, which implies improved electrical conductivity, and the enhanced adsorption of water molecules by the catalyst, both contribute to the improved HER performance. Thanks to these factors, the overpotential of the heterojunction catalyst prepared here is superior to that of most of the reported Co_9_S_8_/MoS_2_-related HER electrocatalysts. 

## 2. Material and Methods

Cobalt nitrate hexahydrate (Co(NO_3_)_2_·6H_2_O, 99.0%), hexamethyl phosphoric triamide (HMTA, 99.0%), and sodium molybdate dihydrate (Na_2_MoO_4_·2H_2_O, 99.0%) were bought from Macklin. Molybdenum disulfide (MoS_2_, 99.0%), cobalt acetate ((CH_3_COO)_2_Co·4H_2_O, 98.0%), thiourea (CH_4_N_2_S, ≥99.0%), and ammonium fluoride (NH_4_F, 99.99%) were bought from Aladdin. The following items were acquired from Sinopharm: potassium hydroxide (KOH, 99.99%), sulfur powder (S powder, 99.0%), ethylene glycol ((CH_2_OH)_2_, 98%), and ethanol (C_2_H_5_OH, ≥99.7%). Pt/C (20 wt%) was supplied by Alfa Aesar. Nafion (5 wt%) was supplied by Sigma-Aldrich. 

The hydrothermal approach was used to create a CoMo-LDH precursor. Firstly, 60 mL deionized water was used to dissolve 2 mmol Co(NO_3_)_2_·6H_2_O, 3 mmol Na_2_MoO_4_·2H_2_O, and 6 mmol HMTA. The prepared solution was then placed into a 100 mL reactor after thorough stirring, and the hydrothermal process was kept at 90 °C for 6 h. Secondly, the precipitates were cleaned with deionized water and alcohol after cooling to room temperature and then dried and centrifuged to obtain the CoMo-LDH precursor.

The prepared CoMo-LDH precursor was transferred to the tubular furnace. S powder was selected as the sulfur source, and 500 mg S powder was placed in an aluminum oxide crucible and put in upstream of the airflow. Next, 50 mg CoMo-LDH was placed in another aluminum oxide crucible and put in downstream of the airflow. The whole reaction process was conducted under the protection of an argon hydrogen (Ar/H_2_) atmosphere. The incoming gas flow rate was set to 40 standard cubic centimeters per minute (sccm). The Co_9_S_8_/MoS_2_ samples were prepared at the following temperatures for 2 h: 350 °C, 450 °C, 550 °C, and 650 °C (the heating rate was 5 °C/min). After the tube furnace had cooled to room temperature, the product in the aluminum oxide crucible located downstream of the gas was removed to obtain the desired sample.

First, 5 mmol cobalt acetate ((CH_3_COO)_2_Co·4H_2_O) and 5 mmol thiourea were dissolved in 30 mL ethylene glycol. The solution was then transferred to a 50 mL high-pressure reactor after 30 min stirring and sealed and held at 200 °C for 48 h. The precipitate was centrifuged once the autoclave had reached room temperature. It was washed three times with deionized water and alcohol and then dried in an oven at 60 °C for eight hours. Co_9_S_8_ was eventually obtained.

### 2.1. Characterizations 

X-ray diffractions were characterized with Cu K radiation using D/MAX 2500 equipment. The morphology and energy dispersive spectrometer (EDS) characterizations were carried out using scanning electron microscopy (Hitachi S4800). A high-resolution transmission electron microscope (JEM-F200) was used to determine the microstructure. A Thermo Scientific K-Alpha apparatus was used to collect the X-ray photoelectron spectra with an Al K source.

### 2.2. Electrochemical Measurements 

The electrochemical properties were determined using a three-electrode electrochemical workstation (CHI 660). A platinum electrode was employed as the counter electrode. A glassy carbon electrode with the sample coated according to the following process was used as the working electrode. First, 5 mg electrocatalyst sample and 80 mL Nafion solution were uniformly dispersed in 1 mL ethanol. The surface of the 3 mm diameter glassy carbon electrode was then splattered with 5 mL electrocatalyst ink. Finally, it was dried at 50 °C to produce the necessary working electrode. An Ag/AgCl (in saturated KCl solution) was chosen for the reference electrode and 1 M KOH (PH = 13.8) was used as the electrolyte. The linear sweep voltammetric (LSV) curves were tested (iR-corrected) at 5 mV/s. Electrochemical impedance spectra Nyquist plots were tested at −400 mV (vs. Ag/AgCl) from 10^5^ Hz to 0.01 Hz. Cyclic voltammetry curves were measured at different scan rates (20 mV/s; 40 mV/s; 80 mV/s; 120 mV/s; 160 mV/s; 200 mV/s). As for the electrochemical stability test, the initial LSV curve (iR-corrected) was tested first, then 1000 cyclic voltammetry cycles were scanned at a sweep rate of 5 mV/s. Another LSV curve (iR-corrected) was tested to compare with the initial LSV curve. The chronoamperometric curve was tested with an overpotential that maintained an initial current density of 10 mA/cm^2^. The test time was set to 50 h. The potential vs. Ag/AgCl electrode was converted to potential vs. RHE according to the following formula: E (RHE) = E (Ag/AgCl) + 0.197 V + 0.059 × pH. The electrochemically active surface area (ECSA) of the catalyst on the glassy carbon electrode was estimated according to the following equation: ECSA = C_dl_/C_s_ × S, where C_dl_ is the electrochemical double-layer capacitance, which can be obtained by testing cyclic voltammetric curves at different sweep rates, C_s_ is the specific capacitance of the sample, which can be estimated as 0.04 mF/cm^2^ in the present work, and S is the surface area of the glassy carbon electrode, which is 0.071 cm^2^ in the present work.

### 2.3. DFT Calculations

The mechanism was revealed by first principle calculations using the Vienna ab initio simulation software [26,27]. In order to optimize the structure and determine the free energy of all structures, the program uses the projected enhancement wave pseudopotential [28] and the generalized gradient approximation of the Perdew–Burke–Ernzerhof (PBE) exchange correlation functional [29]. In the adsorption energy calculation, plane waves basis cutoff energy sets of 500 eV and a Monkhorst-Pack mesh of 2 × 2 × 1 was used in K-sampling. All atom locations were totally relaxed until the residual force on each atom was less than 0.02 eV/−1.20 eV/ of vacuum layer along the z-direction, with the electronic self-consistent iteration set to 10^−5^ eV.

## 3. Results and Discussion

### 3.1. Structural and Morphological Characterization

**Figure 1:**
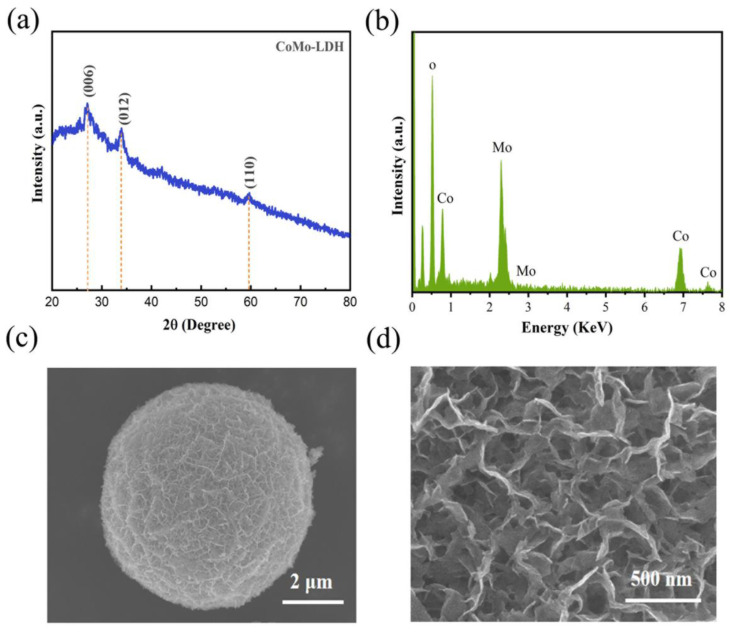
(**a**) XRD pattern; (**b**) EDS analysis; (**c**,**d**) SEM images of CoMo-LDH.

**Figure 2:**
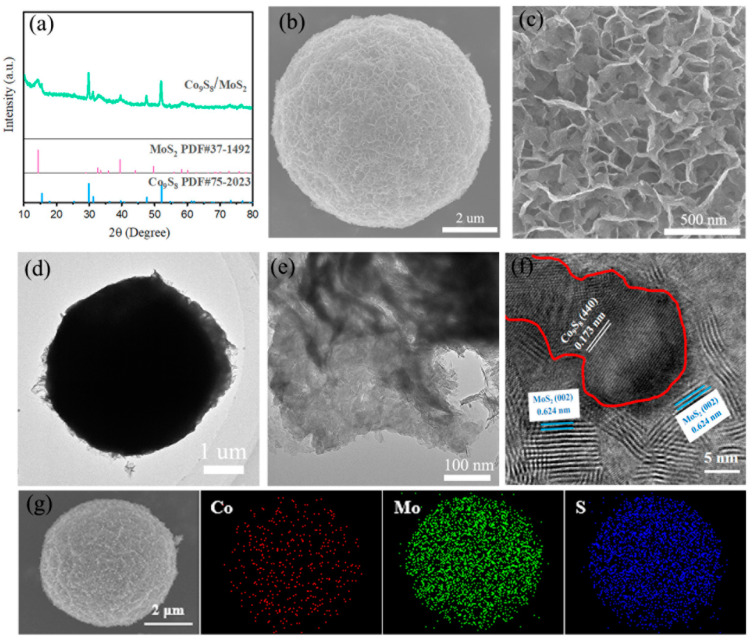
(**a**) XRD patterns of Co_9_S_8_/MoS_2_; (**b**,**c**) SEM images of Co_9_S_8_/MoS_2_ with different scales; (**d**,**e**) TEM images of Co_9_S_8_/MoS_2_ with different scales; (**f**) high-resolution TEM image of Co_9_S_8_/MoS_2_; (**g**) elemental mapping images of Co_9_S_8_/MoS_2_.

**Figure 3:**
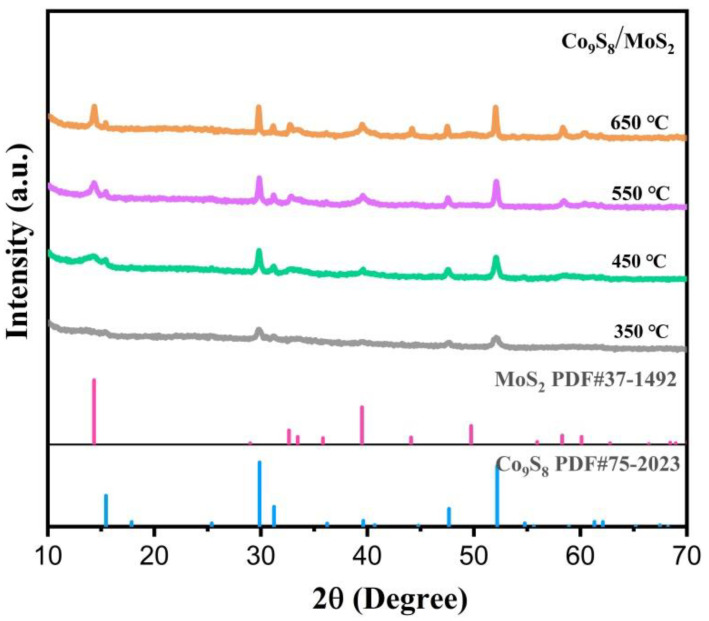
XRD patterns of Co_9_S_8_/MoS_2_ after vulcanization of CoMo-LDH at different temperatures.

**Figure 4:**
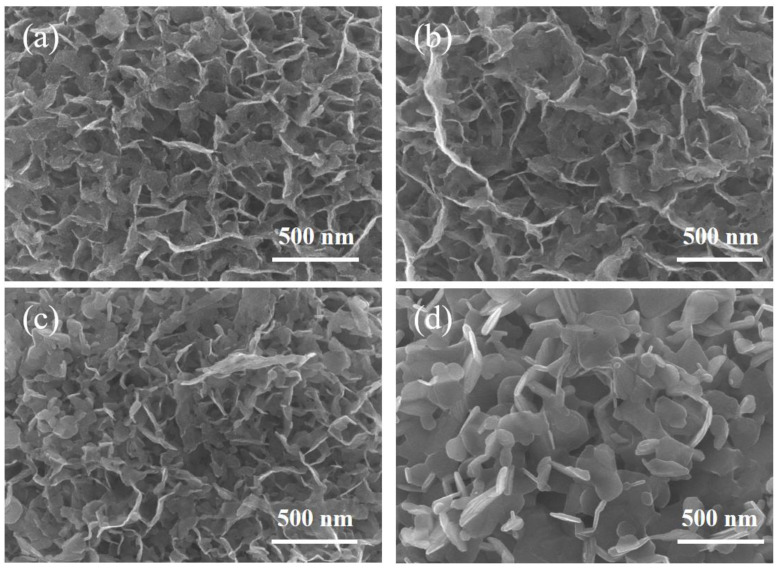
SEM images of Co_9_S_8_/MoS_2_ after vulcanization of CoMo-LDH at different temperatures: (**a**) 350 °C; (**b**) 450 °C; (**c**) 550 °C; (**d**) 650 °C.

**Figure 5:**
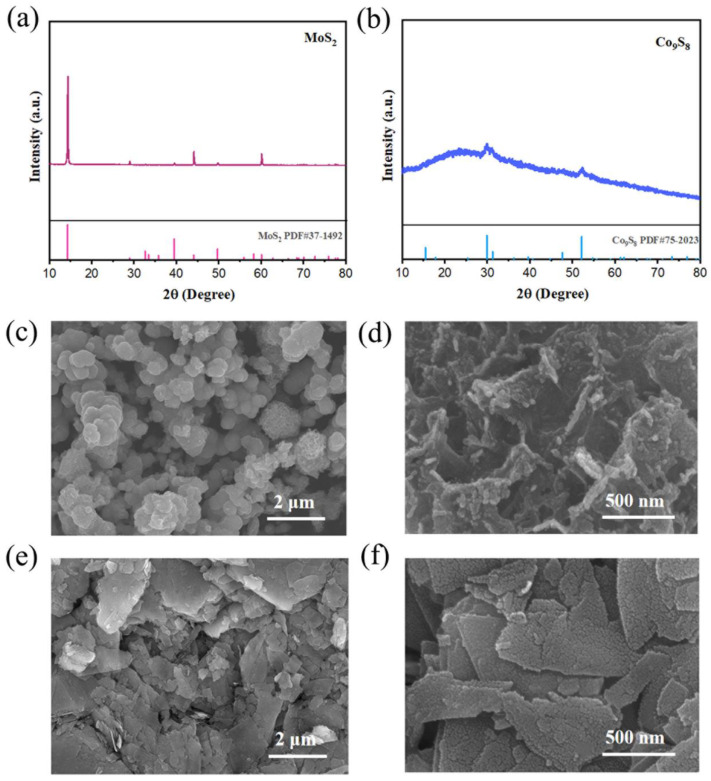
(**a**) XRD pattern of MoS_2_; (**b**) XRD pattern of Co_9_S_8_; (**c**,**d**) SEM images of MoS_2_ with different scales; (**e**,**f**) SEM images of Co_9_S_8_ with different scales.

The synthesis of the Co_9_S_8_/MoS_2_ sample was based on the sulfuration of the precursor CoMo-LDH. The precursor was obtained from a hydrothermal reaction in accordance with the literature [30]. Its composition was confirmed by X-ray diffraction (XRD) (Figure 1a). In addition, energy dispersive spectrometer (EDS) analysis showed that it was composed of three elements—Co, Mo, and O (Figure 1b)—indicating its high purity. The scanning electron microscope (SEM) characterization showed that it presented a morphology of micrometer spheres made of nanosheets (Figure 1c,d). After the CoMo-LDH was vulcanized at 450 °C in a reducing atmosphere, the product was composed of Co_9_S_8_ (PDF-#75-2023) and 2H-MoS_2_ (PDF-#37-1492) (Figure 2a). The product still showed a microsphere morphology consisting of nanosheets (Figure 2b,c), indicating that the sulfidation reaction at a high temperature did not change the overall morphology of the precursor. The presence of many pores between the nanosheets helped to increase the surface area as well as facilitate the migration of electrolytes and bubbles for subsequent electrochemical reactions [31]. To determine how the temperature of the vulcanization reaction affects its products, the precursors were also vulcanized at 350 °C, 550 °C, and 650 °C. XRD characterization showed that the products at these different temperatures were all Co_9_S_8_/MoS_2_ (Figure 3). The diffraction peaks for the product at 350 °C were weaker than those of the other products, indicating its poor crystallinity. SEM characterization showed that the nanosheets became thicker as the reaction temperature increased (Figure 4). From the results of the transmission electron microscopy (TEM) characterization, a distinct microsphere can be observed (Figure 2d). The thin nanosheets can be observed after zooming in on the images (Figure 2e). From the high-resolution TEM image (Figure 2f), clear lattice stripes can be seen. The 0.173 nm stripe corresponds to the (440) crystal plane of the Co_9_S_8_, while the 0.624 nm stripe corresponds to the (002) crystal plane of the MoS_2_. The presence of clear interfaces between them indicates that they form heterojunctions. The EDS elemental mapping revealed that the distribution of Co, Mo, and S in the sample was uniform (Figure 2g). In addition, separate Co_9_S_8_ and MoS_2_ were prepared for a comparative analysis of the properties of the Co_9_S_8_/MoS_2_. Their components as well as the morphology of the nanosheets were confirmed using XRD and SEM characterization (Figure 5).

**Figure 6:**
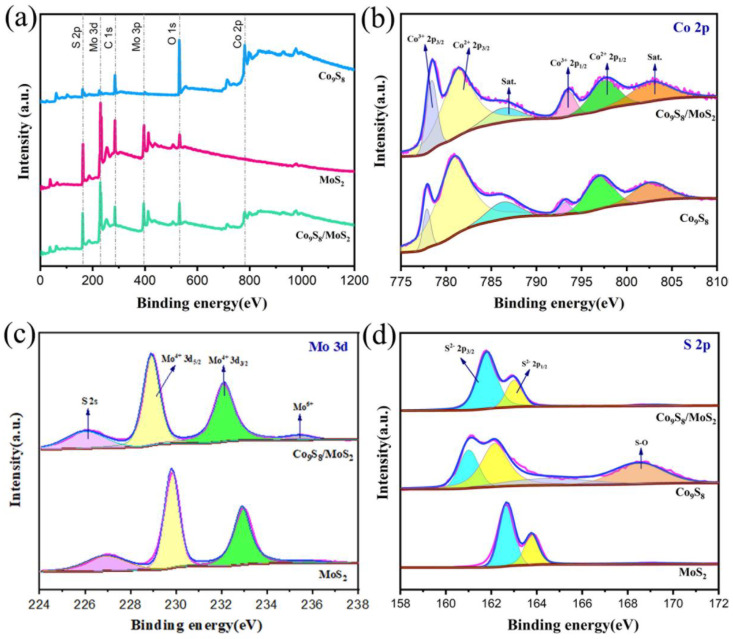
(**a**) XPS survey spectra of Co_9_S_8_, MoS_2_, and Co_9_S_8_/MoS_2_; (**b**) high-resolution Co 2p XPS spectra of Co_9_S_8_ and Co_9_S_8_/MoS_2_; (**c**) high-resolution Mo 3d XPS spectra of MoS_2_ and Co_9_S_8_/MoS_2_; (**d**) high-resolution S 2p XPS spectra of Co_9_S_8_, MoS_2_, and Co_9_S_8_/MoS_2_.

To determine the chemical make-up and elemental states of the samples, X-ray photoelectron spectroscopy (XPS) was employed. The survey spectra of the Co_9_S_8_, MoS_2_, and Co_9_S_8_/MoS_2_ are shown in Figure 6a. The Co_9_S_8_/MoS_2_ sample consists of Co, Mo, S, and O elements, the Co_9_S_8_ sample consists of Co, S, and O elements, and the MoS_2_ sample consists of Mo, S, and O elements. The O elements in the samples are a result of the oxidation of the sample surfaces. The C elements are artificially added to correct the data. For the high-resolution XPS spectra of the Co 2p electrons (Figure 6b), the peaks located near 778.4 eV and 793.6 eV are associated with Co^3+^ and correspond to the Co 2p_3/2_ and Co 2p_1/2_ electrons of the Co^3+^, respectively [32]. The peaks located near 781.5 eV and 797.7 eV are associated with Co^2+^ and they correspond to the Co 2p_3/2_ and Co 2p_1/2_ electrons of the Co^2+^, respectively. In addition, the peaks near 787.7 eV and 803.0 eV are the satellite peaks of the Co 2p electrons. The area of the Co^3+^ peak is significantly larger in the Co_9_S_8_/MoS_2_ compared with the Co_9_S_8_, indicating that a portion of the electrons is transferred from the Co in the Co_9_S_8_/MoS_2_. For the high-resolution XPS spectrum of the Mo 3d electrons (Figure 6c), four peaks were exhibited at 226.0 eV, 228.9 eV, 232.1 eV, and 235.5 eV. They are attributed to the S 2s, Mo^4+^ 3d_5/2_, Mo^4+^ 3d_3/2_, and Mo^6+^ electrons, respectively [33]. Compared with that of the MoS_2_, the Mo^4+^ 3d peak of the Co_9_S_8_/MoS_2_ changes toward lower binding energy, showing that the Mo in the Co_9_S_8_/MoS_2_ has gained some electrons. The appearance of the Mo^6+^ is due to the oxidation of some Mo elements on the surface of the sample. For the high-resolution XPS spectrum of the S 2p electrons (Figure 6d), the peaks between 161 eV and 164 eV can be fitted by two peaks. They correspond to the S^2−^ 2p_3/2_ and S^2−^ 2p_1/2_ electrons, respectively [34]. In the Co_9_S_8_, the peak at 168.5 eV is attributed to the S-O, which results from oxidizing superficial S. According to the aforementioned findings, there is charge transfer between the Co_9_S_8_ and MoS_2_ in the Co_9_S_8_/MoS_2_, which leads to the redistribution of electrons.

### 3.2. Electrocatalytic Performance

**Figure 7:**
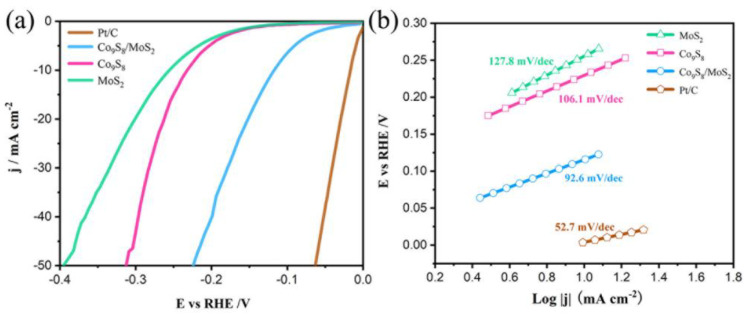
(**a**) Co_9_S_8_, MoS_2_, Co_9_S_8_/MoS_2_ and Pt/C polarization curves in 1 M KOH; (**b**) the corresponding Tafel plots.

To investigate the performance of these samples for the electrocatalytic hydrogen evolution reaction (HER), they were characterized electrochemically. The electrolyte used was 1 M KOH solution. Figure 7a shows the linear scanning voltammetry (LSV) test curves of the samples (which have been 95% iR compensated). The figure shows that the Co_9_S_8_/MoS_2_ exhibits greater HER activity than the Co_9_S_8_ or the MoS_2_ alone at the same current density. The overpotential is only 118 mV at a current density of 10 mA cm^−2^, which is much lower than that of the Co_9_S_8_ at 234 mV or the MoS_2_ at 257 mV. This indicates that there is a synergistic effect between the Co_9_S_8_ and the MoS_2_ which facilitates the hydrogen evolution reaction. In addition, the activity of the Co_9_S_8_/MoS_2_ catalysts fabricated in this work is better than the overpotential values of most relevant heterojunction materials reported in the literature (Table 1) [35,36,37,38,39,40,41,42,43,44]. The Tafel slope of the Co_9_S_8_/MoS_2_ is 92.6 mV dec^−1^, which is lower than that of the Co_9_S_8_ at 106.1 mV dec^−1^ or the MoS_2_ at 127.8 mV dec^−1^ (Figure 7b). The above Tafel slope values also indicate that their HER processes follow the Volmer–Heyrovsky reaction mechanism [45].

**Figure 8:**
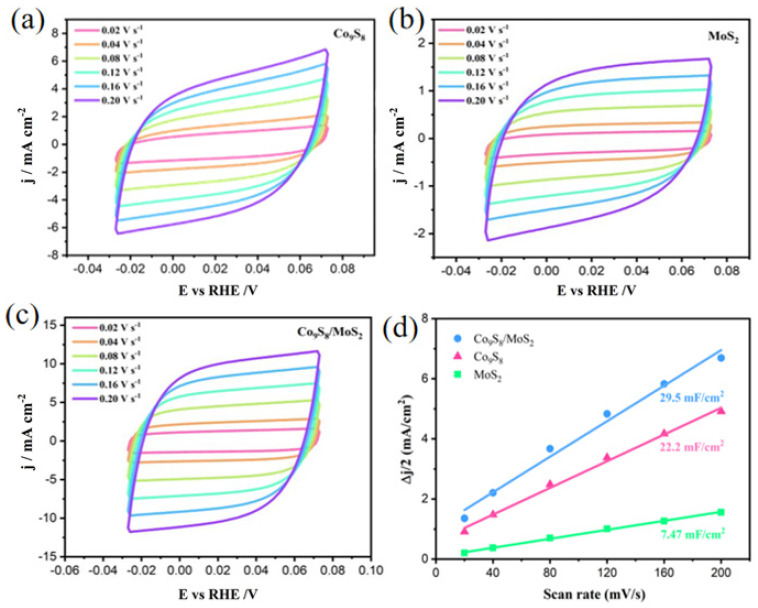
CV curves obtained with different scan rates from 0.02 to 0.20 V s^−1^ in 1 M KOH for (**a**) Co_9_S_8_/MoS_2_, (**b**) Co_9_S_8_, and (**c**) MoS_2_; (**d**) electrochemical double-layer capacitances for Co_9_S_8_, MoS_2_, and Co_9_S_8_/MoS_2_.

**Figure 9:**
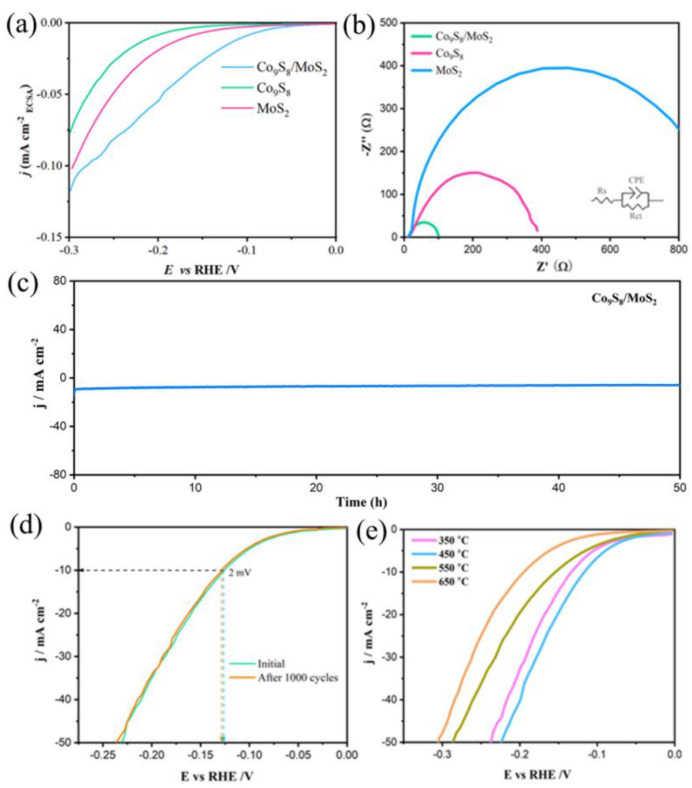
(**a**) Polarization curves normalized to the ECSA of Co_9_S_8_, MoS_2_, and Co_9_S_8_/MoS_2_; (**b**) Nyquist plots for Co_9_S_8_, MoS_2_, and Co_9_S_8_/MoS_2_; (**c**) chronoamperometric curve for Co_9_S_8_/MoS_2_; (**d**) polarization curves of Co_9_S_8_/MoS_2_ before and after 1000 CV cycles; (**e**) polarization curves of Co_9_S_8_/MoS_2_ after vulcanization of CoMo-LDH at different temperatures in 1 M KOH.

To compare the actual electrochemically active surface area (ECSA) of the Co_9_S_8_, MoS_2_, and Co_9_S_8_/MoS_2_ electrodes, a CV test was used in the non-Faraday region to calculate the double-layer capacitance value (C_dl_) of the electrode, since ECSA is proportional to C_dl_. The CV curves of the samples at different sweep rates are shown in Figure 8a–c. The fitted C_dl_ values are shown in Figure 8d. The Co_9_S_8_/MoS_2_ electrode has the largest C_dl_ value (29.5 mF cm^−2^), which is higher than the double-layer capacitance values of both the Co_9_S_8_ (22.2 mF cm^−2^) and the MoS_2_ (7.47 mF cm^−2^). This indicates that the prepared Co_9_S_8_/MoS_2_ electrode has the largest electrochemically active surface area and the most active sites, which is favorable for the HER process. To reveal the effect on the intrinsic activity after the formation of the Co_9_S_8_/MoS_2_ heterojunction, the current density was normalized by the electrochemically active surface area (ECSA). As is shown in Figure 9a, the overpotential of the Co_9_S_8_/MoS_2_ is the smallest among the three at the same current density, which indicates that the formation of the Co_9_S_8_/MoS_2_ heterojunction does improve the intrinsic electrocatalytic hydrogen evolution activity of the single components Co_9_S_8_ and MoS_2_. To further investigate the electrode kinetics of the HER process for the Co_9_S_8_/MoS_2_ heterojunction materials, the electrochemical impedance spectra (EIS) of the Co_9_S_8_, MoS_2_, and Co_9_S_8_/MoS_2_ materials were tested. Typically, the impedance decreases with a decreasing Nyquist curve arc radius. It can be clearly observed from Figure 9b that the Co_9_S_8_/MoS_2_ has a much lower impedance value than the comparative sample. This result indicates that the construction of the Co_9_S_8_/MoS_2_ heterojunction can effectively improve the reaction kinetics of the catalyst. Stability is also an important index for evaluating hydrogen precipitation catalysts. There was no significant increase in current density after 50 h of chronoamperometry tests (Figure 9c). In addition, Figure 9d shows that after 1000 CV tests the overpotential increased by just 2 mV at a current density of 10 mA cm^−2^. These results indicate that the Co_9_S_8_/MoS_2_ heterojunction catalyst has excellent electrochemical stability. In addition, the hydrogen evolution activity was compared for the Co_9_S_8_/MoS_2_ samples obtained at different sulfidation temperatures. As is shown in Figure 9e, the best HER activity was obtained from the sample vulcanized at 450 °C. This may be the result of the combined effect of the crystallinity and morphology of the samples. On the one hand, the sample sulfided at 350 °C is less crystalline, and on the other hand the nanosheets of the products become rougher at higher temperatures (550 °C and 650 °C), which leads to a smaller electrochemically active area. Therefore, 450 °C is the optimal temperature for sulfidation treatment.

### 3.3. DFT Calculations

**Figure 10:**
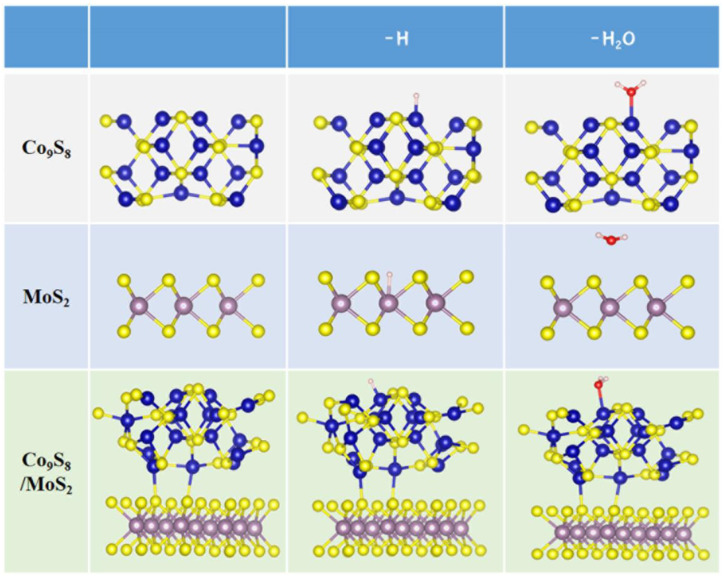
Structure model: pristine; with H adsorption; with H_2_O adsorption for MoS_2_, Co_9_S_8_, and Co_9_S_8_/MoS_2_. Co atoms are represented by blue balls, S atoms are depicted as yellow balls, Mo atoms are depicted as brown balls, H atoms are depicted as white balls, and O atoms are depicted by red balls.

First-principles calculations were carried out to shed more light on the cause of the enhanced activity following the creation of the Co_9_S_8_/MoS_2_ heterostructure. The optimized structural model is shown in Figure 10. 

**Figure 11:**
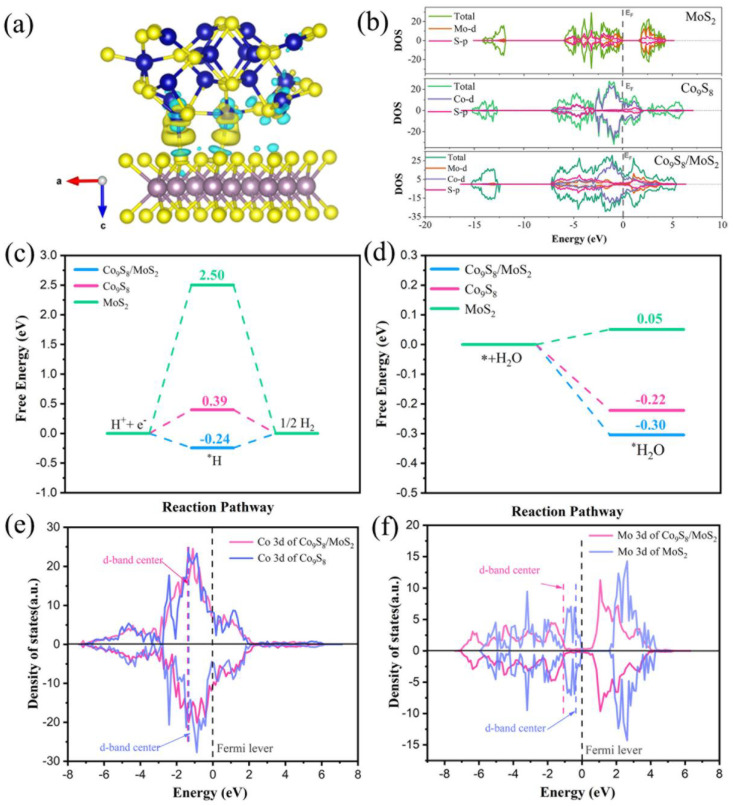
(**a**) Charge density difference for the Co_9_S_8_/MoS_2_ heterointerface with the iso-surface value of the color region being 0.0005 e Å^−3^. The yellow and blue colors indicate the positive and negative charges, respectively. Blue balls represent Co atoms, yellow balls represent S atoms, and brown balls represent Mo atoms. (**b**) DOS curves; (**c**) calculated ΔG_H*_ for the HER; (**d**) calculated water adsorption energy; (**e**) PDOS of Co for Co_9_S_8_ and Co_9_S_8_/MoS_2_; (**f**) PDOS of Mo for MoS_2_ and Co_9_S_8_/MoS_2_.

By calculating the charge density difference of the material, the charge transfer between the different atoms can be visualized. As is shown in Figure 11a, at the interface between the Co_9_S_8_ and MoS_2_, the electron cloud density decreases on the Co_9_S_8_ side and increases on the MoS_2_ side. This indicates that electrons flow from the Co_9_S_8_ to the MoS_2_, and this result is consistent with the results of the XPS characterization. The density of states (DOS) curve (Figure 11b) shows that the MoS_2_ is semiconducting with a forbidden band width of 1.50 eV. The Co_9_S_8_ and Co_9_S_8_/MoS_2_ both exhibit metallicity. The Co_9_S_8_/MoS_2_ has a higher density of states at the Fermi surface than the Co_9_S_8_, indicating that it is more conductive, which would favor the HER process. Hydrogen adsorption free energy (ΔG_H*_) is often used to indirectly reflect the activity of the catalyst [46]. 

A positive ΔG_H*_ value indicates that it is difficult for the catalyst to adsorb hydrogen, whereas a negative ΔG_H*_ value suggests that hydrogen is adsorbing onto the catalyst too tightly. The ideal ΔG_H*_ value is zero. As can be seen in Figure 11c, both the Co_9_S_8_ and the MoS_2_ have positive free energy values for hydrogen adsorption. The ΔG_H*_ value of the Co_9_S_8_ (0.39 eV) is much smaller than that of the MoS_2_ (2.5 eV), indicating that the Co_9_S_8_ has better HER activity than the MoS_2_. The ΔG_H*_ value of the Co_9_S_8_/MoS_2_ is further optimized to only −0.24 eV, which is even closer to zero, indicating that the Co_9_S_8_/MoS_2_ has the best HER activity among the three. The adsorption energy of the catalyst on water molecules is another parameter that can reflect the HER activity [47]. This is due to the fact that the occurrence of the hydrogen evolution reaction in alkaline media first involves the adsorption of water molecules. As is shown in Figure 11d, the adsorption energy of the Co_9_S_8_/MoS_2_ for water molecules is −0.30 eV. It is −0.22 eV for the Co_9_S_8_ and 0.05 eV for the MoS_2_. This indicates that the adsorption of water molecules by the Co_9_S_8_/MoS_2_ is the best among these three. For compounds composed of 3d metals, the position of the *d*-band center is related to the adsorption of hydrogen [48]. Lower adsorption of hydrogen by the catalyst is typically implied by a downward shift of the *d*-band center. The *d*-band centers of the Co_9_S_8_ and MoS_2_ are situated at −1.32 eV and −0.35 eV, respectively, as is shown in Figure 11e,f. They are pushed downward to −1.39 eV and −1.07 eV for the Co_9_S_8_/MoS_2_, respectively. Combined with the previous results concerning the positive ΔG_H*_ values of the Co_9_S_8_ and MoS_2_, the lesser hydrogen adsorption by the catalyst in this case, which is more advantageous to the HER process, is indicated by the *d*-band centers having moved down. The above results indicate that the charge transfer between the Co_9_S_8_ and the MoS_2_ after the formation of the Co_9_S_8_/MoS_2_ heterojunction achieves charge redistribution, thus optimizing the adsorption characteristics of the active sites of the electrocatalyst for hydrogen and water molecules, and thereby realizing the improvement of the electrocatalytic hydrogen evolution performance.

## 4. Conclusions

In summary, we prepared a Co_9_S_8_/MoS_2_ heterojunction by vulcanizing a CoMo-LDH precursor. The vulcanization temperature of 450 °C was suitable to obtain a product with good crystallinity as well as nanosheet thickness. The Co_9_S_8_/MoS_2_ manifests the morphology of a microsphere made of thin nanosheets. Some electrons of the Co_9_S_8_ of the heterojunction were transferred to the MoS_2_, which led to the optimization of the charge distribution. After the formation of the heterojunction, the density of states near the Fermi surface increase, the *d*-band centers of the transition metals shifted downward, and the adsorption of both water molecules and hydrogen by the catalyst were optimized. As a result, the overpotential of the Co_9_S_8_/MoS_2_ at the current density of 10 mA cm^−2^ was only 118 mV, which is better than those of most Co_9_S_8_/MoS_2_-related HER electrocatalysts reported in the literature. This work provides clarification concerning the mechanisms of performance enhancement and morphology regulation of heterojunction electrocatalysts.

## Figures and Tables

**Table 1:** Comparison of HER activity for Co_9_S_8_/MoS_2_ in this work and other reported electrocatalysts (1 M KOH).

Catalysts	Overpotential (mV) @10 mA cm^−2^	Tafel slope (mV dec^−1^)	Reference
Co_9_S_8_/MoS_2_	118	92.6	This work
MoS_2_/FeCo_2_S_4_/CC	161	98.4	[35]
MoS_2_@CoS_2_/G	118	53	[36]
Co(PO_3_)_2_@NPC/MoS_2_	119	142	[34]
MoO_2_/MoS_2_/Co_9_S_8_	160	80	[37]
Co(OH)_2_/1T-MoS_2_	151	94	[38]
S-Co_9_S_8_/MoS_2_/CNFs	122	66	[39]
Co_9_S_8_@MoS_2_/N-doped hollow carbon	126	74.1	[40]
CoS_2_@MoS_2_@NiS_2_	156	81	[41]
Co_9_S_8_-MoS_2_/NF	110	81.7	[24]
Co_9_S_8_/MoS_2_	173	71.5	[42]
Co-MoS_2_	197	61	[43]
Co_9_S_8_/MoS_2_@NSOC	194	118	[44]

## Data Availability

The data presented in this study are available upon request from the corresponding author.
